# A Randomized, Double-Blind Placebo Control Study on the Effect of a Blood Flow Restriction by an Inflatable Cuff Worn around the Arm on the Wrist Joint Position Sense in Healthy Recreational Athletes

**DOI:** 10.3390/jcm12020602

**Published:** 2023-01-11

**Authors:** Aleksandra Królikowska, Klaudia Kusienicka, Ewa Lazarek, Łukasz Oleksy, Robert Prill, Anna Kołcz, Maciej Daszkiewicz, Dariusz Janczak, Paweł Reichert

**Affiliations:** 1 Ergonomics and Biomedical Monitoring Laboratory, Department of Physiotherapy, Faculty of Health Sciences, Wroclaw Medical University, 50-367 Wroclaw, Poland; 2 Oleksy Medical & Sports Sciences, 37-100 Łańcut, Poland; 3 Department of Physiotherapy, Faculty of Health Sciences, Jagiellonian University Medical College, 31-008 Kraków, Poland; 4 Center of Orthopaedics and Traumatology, University Hospital Brandenburg/Havel, Brandenburg Medical School Theodor Fontane, 14770 Brandenburg a.d.H., Germany; 5 Faculty of Health Sciences Brandenburg, Brandenburg Medical School Theodor Fontane, 14770 Brandenburg a.d.H., Germany; 6 Department of Vascular, General, and Transplantation Surgery, Faculty of Medicine, Wroclaw Medical University, 50-367 Wroclaw, Poland; 7 Department of Trauma Surgery, Clinical Department of Trauma and Hand Surgery, Faculty of Medicine, Wroclaw Medical University, 50-367 Wroclaw, Poland

**Keywords:** blood flow restriction therapy, physical therapy modalities, rehabilitation, sports and recreational facilities, sports medicine, upper extremity, wrist joint

## Abstract

The number of blood flow restriction (BFR) training practitioners is rapidly increasing, so understanding the safety issues associated with limb occlusion is strongly needed. The present study determined the effect of BFR by an inflatable cuff worn around the arm on the wrist joint position sense (JPS) in healthy recreational athletes. In the prospective randomized, double-blind placebo control study, sixty healthy right-handed recreational athletes aged x = 22.93 ± 1.26 years were assigned to groups of equal size and gender rates: BFR, placebo, and control. The active wrist JPS was assessed in two separate sessions using an isokinetic dynamometer. The first assessment was performed with no cuffs. In the second session, a cuff with a standardized pressure was worn on the examined limb in the BFR group. In the placebo group, the cuff was uninflated. A between-session comparison in each group of collected angular errors expressed in degrees was carried out. The angular error in the BFR group was larger during the second measurement than the first one (*p* = 0.011–0.336). On the contrary, in the placebo (*p* = 0.241–0.948) and control (*p* = 0.093–0.904) groups, the error value in the second session was comparable or smaller. It was determined that BFR by an inflatable cuff around the arm impairs the wrist position sense. Hence, BFR training should be performed with caution.

## 1. Introduction

Blood flow restriction (BFR) in a controlled form of vascular occlusion using an external tourniquet continues to gain attention in sports and rehabilitation. Based on current literature, BFR has been used in conjunction with resistance training [1], aerobic exercises [2], or sport-specific training [3], and is then defined as BFR training. The most significant popularity of BFR was based on evidence that low-intensity aerobic training with BFR increases muscle size and strength and improves the anaerobic capacity on a level comparable to high-intensity training with no BFR [4]. The use of BFR in adults with obesity has also been supported [5]. The literature also advises using BFR during rest periods to prevent muscle atrophy or to use occlusion during training in order to reduce exercise-induced delayed-onset muscle soreness [6]. Hence, it may also be named BFR therapy or considered a physiotherapeutic modality.

The external tourniquet used for BFR purposes is placed above the exercised segment or directly on it. In this method, the occlusive device restricts the blood flow in the upper or lower limbs throughout the muscle contraction cycle and rest period or only during rest intervals [5]. This results in a partial restriction of the arterial supply to the muscle, but the greatest extent of the restriction is on the venous outflow from the muscle [4]. On the market, there is a large variety of BFR devices [7,8]. A pneumatic cuff with a pressure-controlling device that precisely applies the target pressure is often used in experimental and clinical settings [9,10]. However, different kinds of elastic bands or belts are also used [11,12].

The proprioception of the joint plays a crucial role in maintaining its stability and normal coordination during movement and in protecting against injurious movements [13]. The term proprioception in the current literature most commonly addresses the conscious proprioception belonging to somatosensory senses composed of kinesthesia, the sensation of the joint position, and the sense of the tensile or force [14]. Contrary, unconscious proprioception refers to neuromuscular control. The conscious sensation of joint position is defined as joint position sense, JPS, which constitutes a separate entity from kinesthesia, the conscious sensation of joint motion [15]. The two sensations also differ in their central processing and interpretation in the brain [16].

As the JPS is of crucial importance in the proper wrist joint dynamic control and function [17,18,19], it seems reasonable and justified to gain knowledge concerning the effect of the occlusion used for BFR training purposes for the sensation of the position of the joint. This information would be highly significant in light of the safety issues of BFR training. So far, the effect of BFR on the JPS seems to have been raised only in the study of Yamada et al. [12]. It was concluded that walking with BFR applicated with elastic bands on lower limbs might be safely performed without increasing the risk of injury as it does not impair the knee JPS. However, it has to be noted that the knee JPS was measured after BFR walking. Of course, it is essential to determine whether proprioception is impaired just after the training. However, in light of safety issues, it is far more crucial to know whether the sense of joint positioning is affected when the BFR is applicated. The proprioception impairment induced during occlusion would be of high interest when performing BFR in conjunction with aerobic exercises or sport-specific training.

Therefore, the present study aimed to determine the effect of a blood flow restriction by an inflatable cuff worn around the arm on the wrist joint position sense in healthy recreational athletes. Based on existing knowledge, it was hypothesized that the sensation of the positioning of the wrist joint would neither be positively nor negatively affected by the occlusion of the arm.

## 2. Materials and Methods

The study was carried out in 2021–2022 in the Ergonomics and Biomedical Monitoring Laboratory in the Department of Physiotherapy and the Department of Trauma Surgery Faculty of Medicine, Wroclaw Medical University in Wroclaw, Poland.

### 2.1. Design of the Study

The prospective interventional study used a randomized, parallel, double-blind placebo control study design. The primary outcome was the change in wrist JPS. The change was determined by comparing the results obtained at baseline (1st measurement) and during the intervention (2nd measurement). An intervention was the application of BFR via an inflatable cuff worn around an arm. The study intervention was compared against an inactive intervention with no treatment value given to the control group and no intervention. The time between the baseline measurement and the second measurement exceeded 90 min.

### 2.2. Participants

The volunteers were recruited between October 2021 and February 2022 from the students at Wroclaw Medical University. The study was carried out in a group of the first 60 recreational athletes, including 30 males and 30 females, who, based on a taken history, met all of the inclusion criteria and did not meet any of the exclusion criteria. The inclusion criteria were as follows: age ranging from 20 to 30 years; dominant right limb determined to be that generally used for daily activities and writing; physical activity level: moderately active (training 2–3 times a week for a minimum of one hour; average 208 min of activity per week) or very active (training 3–4 times a week for a minimum of one hour; average 420 min of activity per week); engagement or participation in sports or fitness activities for recreational purposes; lack of diagnosed circulatory insufficiency; absence of any symptoms suggesting circulatory insufficiency; lack of any diagnosed systematic disease; absence of pain or any other symptoms in the upper limbs or cervical or thoracic spine; absence of injury and/or disease in the upper limb(s) and cervical or thoracic spine in the history. On the contrary, the following criteria were considered as excluding: age less than 20 years or more than 30 years; dominant left limb determined to be that generally used for daily activities and writing; physical activity level: inactive or sedentary (activity solely related to household duties; average 140 min of activity per week) or extremely active (a professional athlete who trains a minimum of 6 h a week or a hard physical worker; average of 560 min of activity per week); no engagement or participation in sports or fitness activities at all or engagement or participation in sports or fitness activities for other than recreational purposes; diagnosed circulatory insufficiency or presence of any symptoms suggesting circulatory insufficiency; diagnosed systematic disease; the presence of pain or any other symptoms in the upper limbs or cervical or thoracic spine; injury and/or disease in the upper limb(s) and cervical or thoracic spine in the history.

**Figure 1:**
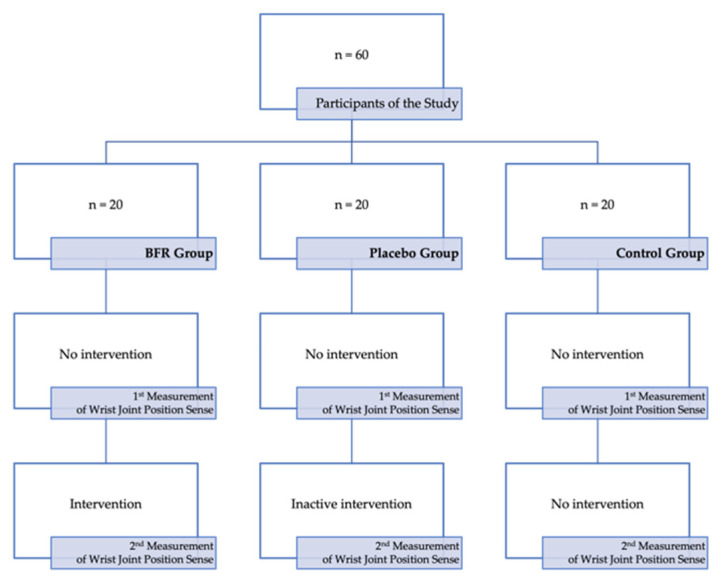
The scheme of the present study.

The studied sample was divided into three equal groups: the BFR group, to which the intervention was applied, the placebo group, with inactive intervention with no treatment value, and the control group, with no intervention, as presented in Figure 1.

Allocation was concealed using sealed opaque envelopes. Stratified distribution using gender was employed in the study. The randomization was carried out by a statistician using a randomization table created by computer software. The person who determined if a participant was eligible for inclusion was unaware, when this decision was made, to which group the participant would be allocated. Blinding was applied to participants of the study, the person assessing the outcomes (examiner), and the person analyzing the results (statistician).

Before the examination, in all studied participants, the body mass (kg) and body height (m) were measured, and, consecutively, body mass index (kg/m^2^) was calculated.

### 2.3. Blood Flow Restriction

**Figure 2:**
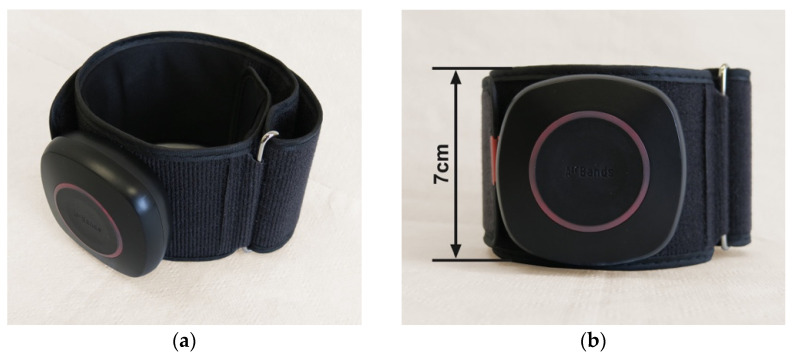
One of the two blood flow restriction training cuffs used for the study purposes: (**a**) the front view of the cuff; (**b**) the side view from above.

For the study purposes, wireless BFR cuffs—more precisely, Airbands (VALD Health, VALD Pty Ltd., Newstead QLD, Australia) —were used (Figure 2). Two dedicated upper limb cuffs with a length of 25–45 cm and width of 7 cm, separately for the right and left arm, were used.

The Airbands cuffs consist of a PVC module attached to a band. The bands come in a pair of sizes separately for upper limbs and lower limbs.

**Figure 3:**
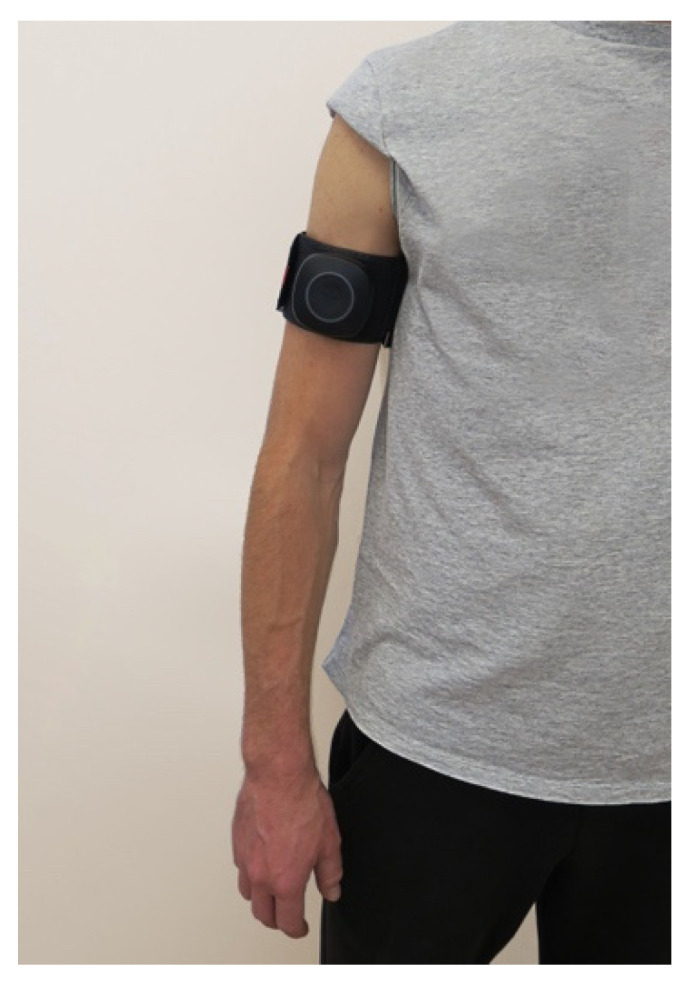
Placement of the BFR cuff on the right arm directly over the deltoid tuberosity.

Just before the second measurement of the wrist JPS in the BFR and placebo groups, the arm cuff was placed directly over the deltoid tuberosity of examined limb, starting with the right one, as presented in Figure 3. The examiner ensured that the module was facing forward and that the logo could be read the right way up. Consecutively, the cuff was fastened in the correct position by wrapping the band through the metal loop. Then, the examiner ensured that there enough room was left under the cuff for two fingers between the skin and the cuff. Next, the cuff was paired with the AirBands App installed on the tablet (Galaxy Tab S7 SM-T870, Samsung Electronics Co., Ltd. 129, Samsung-ro, Yeongtong-gu, Suwon-si, Gyeonggi-do, Republic of Korea) via Bluetooth. After pairing with the software, the cuff was calibrated. During calibration, the cuff was inflated to automatically detect the maximal (100%) limb pressure. Throughout the JPS assessment of the limb, 50% of the maximal pressure detected during the calibration was applied in the BFR group. The pressure was set according to the manufacturer’s guidelines. At the same time, in the placebo group, the cuff stayed uninflated. After examining the right limb, the cuff pressure was deflated in the BFR group. Next, the procedure was repeated on the left limb. The intervention was proceeded by an investigator trained and experienced in BFR training.

### 2.4. Joint Position Sense Assessment

**Figure 4:**
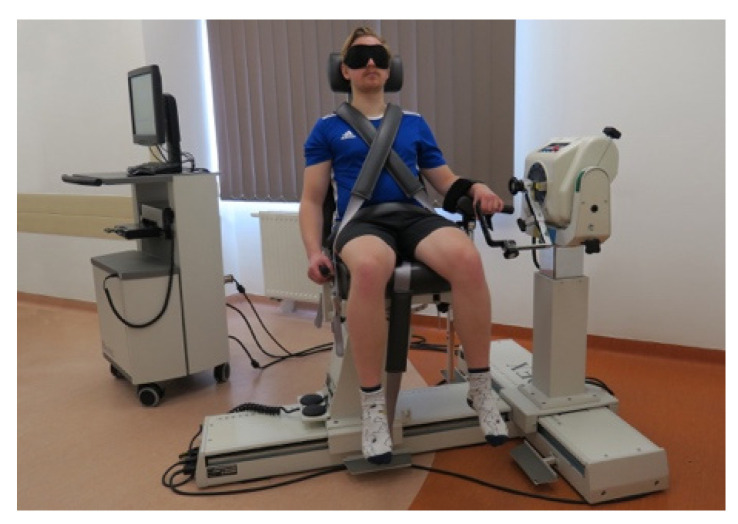
The position of the participant of a study on an isokinetic dynamometer.

The wrist JPS was measured using an active joint position reproduction test performed on an isokinetic dynamometer—more precisely Biodex System 4 Pro (Biodex Medical Systems, Inc., Shirley, NY, USA)—as presented in Figure 4. The participants wore comfortable sports outfits and black masks covering their eyes during the measurement. The measurement was performed in a seated position with the forearm of examined limb flexed at the elbow at 90° and pronated.

**Figure 5:**
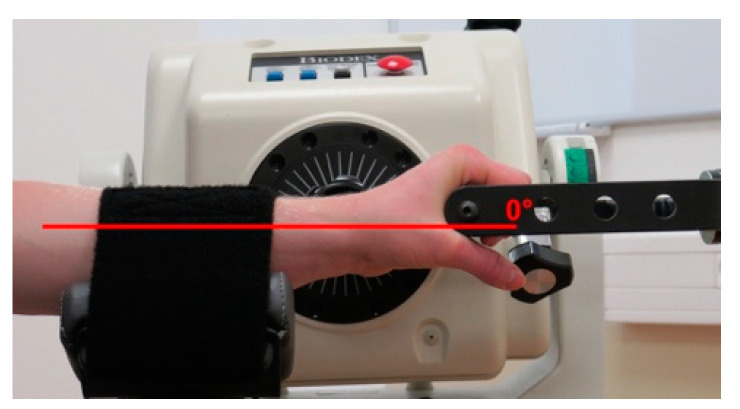
The starting position for the wrist joint position sense measurement.

The starting wrist position was 0°, as presented in Figure 5.

**Figure 6:**
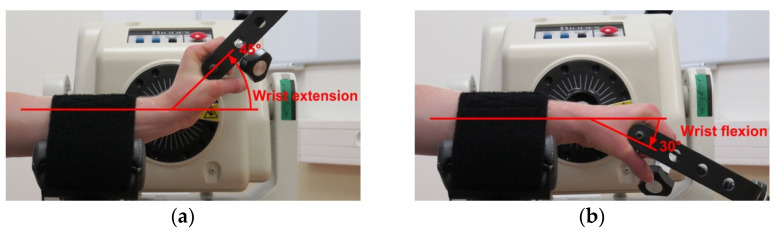
The two predefined target positions during the wrist joint position sense assessment: (**a**) the target position of a 45° wrist extension; (**b**) the target position of a 30° wrist flexion.

At first, the examined wrist was passively moved to a predetermined target position for the active joint position assessment. The wrist remained in this position for ten seconds so that the participant could remember the specific position, and, consecutively, the wrist was passively moved to starting position. After staying at the starting position for three seconds, the participant was asked to move the examined wrist to match the target position actively. The position considered by the participant as the target one was registered by the device. Two target positions were examined in the same way—more precisely, a 45° wrist extension and 30° wrist flexion—as presented in Figure 6. At each position, two repetitions were performed. The measurements were carried out bilaterally, starting with the right upper limb, and by an examiner experienced in using an isokinetic dynamometer.

The collected parameter was the absolute difference between the target and actively replicated position, defined as an absolute angular error expressed in degrees (°). For further analysis, the arithmetic mean from two repetitions for each target position was calculated and expressed as the mean absolute angular error (°), with higher values indicating worse JPS.

It should be highlighted that JPS assessment using position reproduction test is commonly used for research purposes [20,21,22]. The reliability of the wrist JPS measurement using an isokinetic dynamometer has been indicated as good with an intraclass correlation coefficient, ICC, ranging from 0.763 for the nondominant limb to 0.821 [23].

### 2.5. Statistical Analysis

SPSS Statistics Version 28.0.1.0 (142) (IBM^®^ SPSS^®^ Statistics, Armonk, NY, USA) and Microsoft Office Excel 365 (Microsoft Corporation, Redmond, WA, USA) were used for statistical analysis.

In an a priori pilot study, an average negative influence BFR compared to placebo and no intervention, with an effect of between-group factors of 0.27, was observed. Based on this, a sample size calculation with linear Eta-square of 0.25, 1-ß of 0.95, α = 0.05, three groups, and two measurements was performed with G*Power, resulting in an estimated necessary sample size of 57 participants. To be sure that the minimum number of participants would participate in both JPS measurements, 60 participants were qualified for the study.

The arithmetic mean (x) and standard deviation (±) were calculated for the age, body mass, body height, and BMI As according to the performed Shapiro–Wilk test. The features were normally distributed, and the between-group comparison in terms of age, body mass, body height, and BMI was carried out using the one-way analysis of variance (ANOVA).

The arithmetic mean and the 95% confidence interval (CI) were determined for mean absolute angular error for each target position and separately for the right and left upper limbs. For each studied group, the comparison of values of mean absolute angular errors obtained during the baseline measurement separately in the right and left upper limb and the values obtained during the second measurement was carried out. Firstly, the Shapiro–Wilk test was performed. In cases where both features being compared revealed the normal distribution, the parametric *t*-test for dependent samples was used. On the contrary, when at least one of two features being compared did not reveal normal distribution, a nonparametric *t*-test for dependent samples was used; more precisely, the Wilcoxon test.

For the between-group analysis purposes, the change in mean absolute angular error for each target position and separately for the right and left upper limbs was calculated by subtracting the value obtained during the first measurement from the value obtained during the second measurement. The higher values indicated worsening JPS, whereas the lower values indicated better JPS. The arithmetic mean and 95% CI were determined for the studied feature. The Shapiro–Wilk test showed the normal distribution of the studied features, and a between-group comparison was carried out using the one-way analysis of variance (ANOVA). The statistical significance was set at *p* < 0.050.

## 3. Results

There were no losses and no exclusions after randomization. All 60 participants took part in both measurements. No harm or unintended effects were noted in any of the participants. Nobody was excluded from the analyses. There were no missing data. The three studied groups were comparable in terms of age (*p* = 0.128), body mass (*p* = 0.660), body height (*p* = 0.354), and BMI (*p* = 0.571), as presented in Table 1.

In the BFR group, the values of the mean absolute angular error measured between the reproduced position and the target position set at a 45° wrist extension were larger during the intervention than at the baseline, as presented in Table 2. The change was not statistically significant (*p* = 0.183–0.271). On the contrary, in both the placebo and control groups, the mean absolute angular error values were smaller during the second measurement than during the first. The differences were also not statistically significant (*p* = 0.093–0.477).

As presented in Table 3, the mean absolute angular error values obtained for the target position set at a 30° wrist flexion were statistically significantly larger (*p* = 0.011–0.019) during the intervention than with no intervention. Again, in the placebo and control groups, the mean absolute angular error values were comparable or smaller during the second measurements. The differences were not statistically significant (*p* = 0.241–0.948).

**Figure 7:**
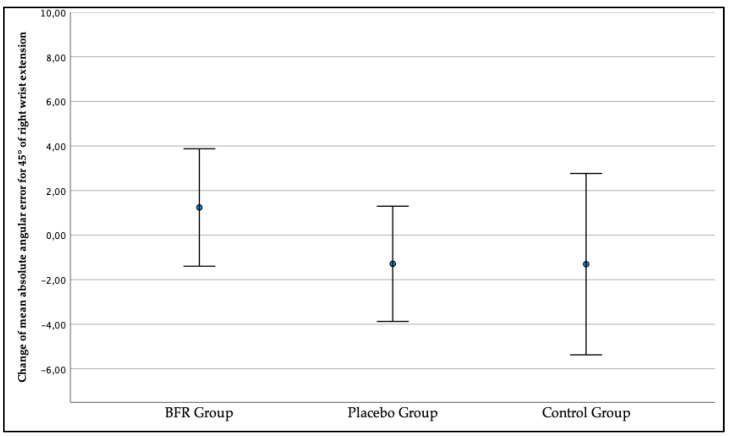
The change in the three studied groups of mean absolute angular error of the right wrist joint position sense for the target position of a 45° wrist extension.

The change in mean absolute angular error amounting to x = 1.24° (−1.39, 3.88) indicated a worsening of the right wrist JPS for the target position of a 45° wrist extension in the BFR group when comparing the first measurement to the second measurement as presented in Figure 7. Contrary, in the placebo group and control group, the right wrist JPS improved in the second measurement as the change consecutively exceeded x = −1.29° (−3.87, 1.30) and x = −1.30° (−5.37, 2.77).

**Figure 8:**
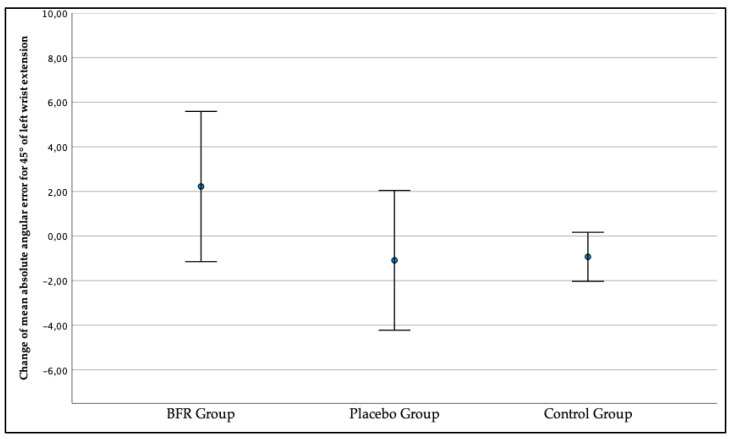
The change in the three studied groups of mean absolute angular error of the left wrist joint position sense for the target position of a 45° wrist extension.

In addition, as presented in Figure 8, the left wrist JPS for the target position of a 45° wrist extension got worse during the second measurement in the BFR group, x = 2.22° (−1.14, 5.59) and improved in the placebo group, x = −1.09° (−4.22, 2.05), and control group, x = −0.93° (−2.03, 0.17). However, the between-group differences in mean absolute angular error changes were not statistically significant for both right (*p* = 0.399) and left (*p* = 0.138) limbs.

**Figure 9:**
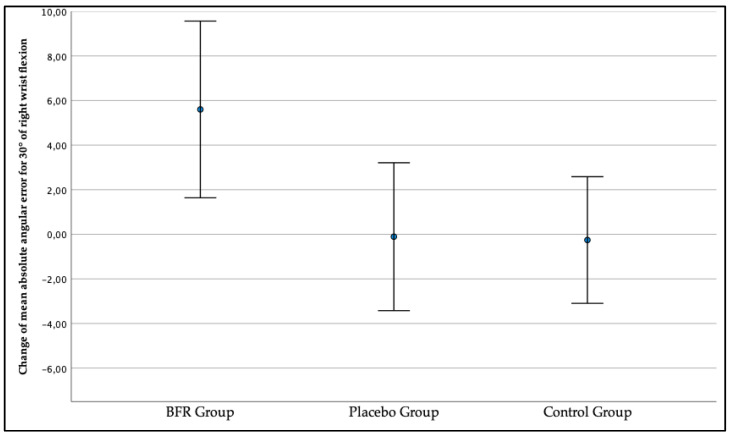
The change in the three studied groups of mean absolute angular error of the right wrist joint position sense for the target position of a 30° wrist flexion.

As presented in Figure 9, the right wrist JPS for the target position of a 30° wrist flexion got worse in the second measurement compared to the first measurement in the BFR group as the change in mean absolute angular error exceeded x = 5.60° (1.64, 9.57). In the placebo and control groups, the right wrist JPS improved, with the change exceeding x = −0.11° (−3.42, 3.21) and −0.26 (−3.09, 2.58). The between-groups comparison revealed statistically significant differences (*p* = 0.020).

**Figure 10:**
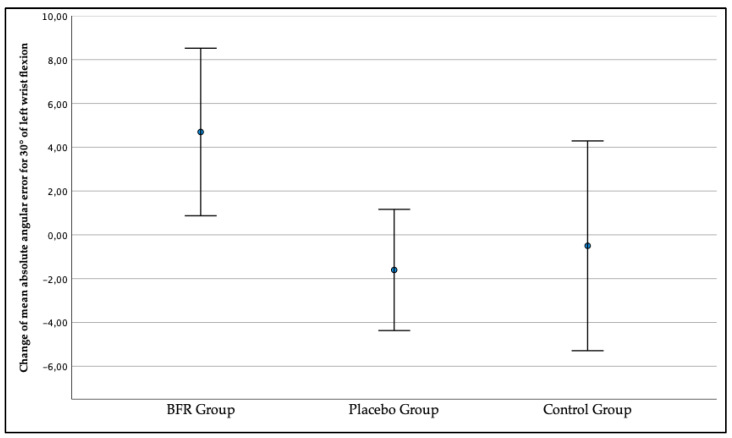
The change in the three studied groups of mean absolute angular error of the left wrist joint position sense for the target position of a 30° wrist flexion.

In addition, as presented in Figure 10 the left wrist JPS for the target position of 30° in the BFR group got worse during the second measurement compared to the first measurement, with the change amounting to x = 4.70° (0.87, 8.52). Conversely, the left wrist JPS improved during the second measurement as the change exceeded x = −1.60 (−4.37, 1.17) in the placebo group and x = −0.50 (−5.29, 4.28) in the control group. According to the performed ANOVA test, the between-group differences were statistically significant (*p* = 0.044).

## 4. Discussion

The present randomized, double-blind placebo control study aimed to evaluate the effect of BFR by an inflatable cuff worn around the arm on the wrist JPS in healthy recreational athletes. The effect was firstly determined by comparing the values of the mean absolute angular error obtained in the three studied groups during the measurements of wrist JPS. Only in the BFR group were the values larger in the second measurement, indicating a worsening of the joint position sensation when BFR was applied. In contrast, in the placebo and control groups, the mean absolute angular error values were comparable or smaller in the second measurement compared to the first. Secondly, the effect of BFR was assessed based on the change in the mean absolute angular error for each target position when comparing measurements without and with BFR. In the case of the BFR group, a change for the worse was observed, and, in the other two groups, the change indicated no effect or an improvement in the wrist JPS. Therefore, the primary finding of the present study was that, in healthy recreational athletes, BFR by an inflatable cuff around the arm impairs the wrist JPS, and our hypothesis that the sensation of the positioning of the wrist joint would not be positively nor negatively affected by the occlusion of the arm was rejected.

The current literature lacks studies on joint proprioception during BFR training, even though it is essential to consider the safety issues of using BFR. So far, it has been assumed that the risks of performing BFR training are comparable to those of traditional training and include, for example, blood clotting and muscle damage [4,24]. Regarding proprioception, in the literature, we can find one study conducted by Yamada et al. on the effect of low-intensity aerobic exercise—more precisely, walking with a BFR applicated using an elastic band—on knee proprioception [12]. Yamada et al. assessed changes in knee joint absolute angular errors determined using an active joint position reproduction test before and after the exercise protocol. On two separate visits, healthy young adults performed walking on a treadmill with or without BFR. It was concluded that the application of BFR in addition to the exercise did not affect knee proprioception; accordingly, BFR walking can be safely utilized for healthy individuals. In our opinion, it is evident that the effect of BFR training on the sense of the joint is of significant importance. However, from the point of view of safety, it is much more essential to assess possible disorders of the sensation of joint position during training under occlusion conditions. Our results are opposite to Yamada et al., but we measured the JPS during occlusion, not after occlusion. In the study by Yamada et al., the BFR belts were removed after the exercise and before the JPS measurement [12]. Even if the proprioception impairment would be only momentary—more precisely, only during the occlusion—it might increase the risk of injury when performing blood-flow-restricted aerobic exercises or BFR sport-specific training. Of course, it has to be mentioned that, in our study, we addressed the wrist joint, whereas Yamada et al. assessed the knee joint. Therefore, future studies on the effect of occlusion on the knee and the elbow and ankle position sense should be determined.

The mentioned study conducted by Yamada et al. included the effect of BFR walking not only on knee joint proprioception but also on muscle fatigue, which was determined by changes in the power outputs [12]. Yamada et al. found no evidence that BFR walking induces fatigue, supporting the results of a previous study by Loenneke et al. [25]. It was correctly highlighted in the article of Yamada et al. that the observed lack of proprioception impairment might be related to the absence of muscular fatigue. It is well known that the sensorimotor control sense provides the body with a distinct ability to recognize the joint position via input muscle mechanoreceptors and from the skin [14,15]. The primary regulatory mechanoreceptor responsible for the JPS is the muscle spindle [14,15]. It has been proven that muscle spindles are vulnerable to muscle fatigue; hence, the rigorous activity of the wrist [26] and other joints [27,28,29] adversely affects the sensation of the joint position. Accordingly, two critical topics still need to be addressed in future studies. Firstly, the general topic of muscle fatigue under occlusion, and secondly, the effect of BFR training on proprioception, but when the muscle fatigue during exercise is present.

The joint position is a component of proprioception, being crucial in joint stability, coordination, and protection against injurious movements. The definition of proprioception covers the detecting and consecutive processing of the stimulus followed by initiating a reactive output through the neuromuscular system. Proprioception addresses the conscious and unconscious perception of movement, posture, and joint position [14]. The evaluation of the JPS addresses the ability of the examined person to accurately reproduce a specific joint angle that is achieved using a so-called position reproduction test. The joint position reproduction test protocol used for the present study is an adjustment method, where the participant is asked to reproduce or match the previously experienced reference joint position [30]. Joint position reproduction testing is conducted under either passive or active conditions for criterion and reproduction movements.

Furthermore, joint reproduction testing may involve either ipsilateral or contralateral limb movements. The present study used the ipsilateral type as the participant needed to remember the target position and reproduce the position using the same limb. Regarding contralateral testing, we distinguished two types of target position reproduction. One method is very similar to the ipsilateral testing method. The familiarization with the target position proceeds in one limb consecutively, the participant returns to the starting position, and the contralateral limb reproduces the target position. In other words, the participant needs to remember the target position and use the opposite limb to reproduce it. In the second method, once one joint is moved to the target position, it remains in it, and the contralateral limb consecutively reproduces this position [30]. The gold standard device for assessing the JPS is an isokinetic dynamometer. Robotic devices are also used for wrist joint position assessment [31,32]. It is worth mentioning, to some limited extent, that the test can be easily performed using commonly available tools. For example, the wrist JPS using a standard goniometer was standardized by Karagiannopoulus et al. [33,34]. An interesting tool used for wrist JPS training so far is the so-called joint position sensometer [35], which seems to have the potential to also be an assessment instrument. Another instrument worthy of notice is a device developed by Gay et al. [36] using a methodology designed to minimize extraneous factors and isolate the muscle and joint inputs. In their study, Van de Winckel et al. used a valuable JPS measuring instrument called the bimanual wrist manipulandum [36]. Other authors used wearable sensors [37]. The wrist JPS could also be assessed using novel devices and systems designed for motion analysis [38].

Since BFR training has been used primarily in sports, the target population in the present study was healthy recreational athletes. The target population was directly represented by the trial [39]. Estimating the effects of arm occlusion in populations different from healthy recreational athletes is not possible. Since the current literature pays more and more attention to the use of BFR in rehabilitation, a similar study should be conducted in the future on patients with the following trauma, as we know that they already present deficits in the JPS [19]. We can speculate that, in those patients, BFR could impair proprioception even more prominently, but until it is investigated, it remains discussable.

The most considerable limitation of the present study was that the wrist JPS assessment was always performed in the same order. In the present study, the first measured target position was a 45° wrist extension, and the second measured target position was a 30° wrist flexion. The more significant JPS impairment was noted for the target position of a 30° wrist flexion. However, it is impossible to determine if the more significant JPS impairment was noted because of the particular joint position or because of the longer duration of occlusion. Therefore, there is a strong need for establishing a trial including a random order of target positions in the future.

The authors put much effort into avoiding any potential sources of bias [40]. Therefore, they used a prospective design of an interventional study. Secondly, the allocation was concealed using sealed opaque envelopes. The stratified distribution using gender was employed in the study to be sure that, in all studied groups, the gender ratio would be exactly the same. Blinding was applied to participants of the study, the person assessing the outcomes (examiner), and the person analyzing the results (statistician). Furthermore, the researcher determining if a participant was eligible for inclusion was unaware, when this decision was made, to which group the participant would be allocated. In addition, the group of researchers preparing the study and the manuscript contained specialists in different areas related to BFR; more precisely, physical therapists, a sports medicine specialist, and an orthopedic surgeon, as well as a vascular specialist.

The gained knowledge on the effect of BFR by an inflatable cuff worn around the arm on the wrist JPS in healthy recreational athletes should influence clinicians and trainers to pay special attention when introducing BFR to the training or rehabilitation program. The topic also seems important because of a rapid increment in the number of BFR training practitioners. Different types of bands and cuffs are available, and it seems like the safety issues of their usage are not broadly discussed. Joint proprioception is based upon the integration of afferent signals from proprioceptors in different joint structures and is also affected by signals from outside the joint; for example, vestibular organs, the visual system, and cutaneous and proprioceptive receptors from other parts of the body [41]. Hence, if the joint proprioception is impaired because of BFR, it seems reasonable to advise controlling the performed movements with applicated BFR using, for example, visual feedback. Athlete or patient education on BFR usage should also be improved.

## 5. Conclusions

In healthy recreational athletes, blood flow restriction by an inflatable cuff worn around the arm impairs the wrist joint position sense. Therefore, special care should be taken during BFR training. However, a strong need for future studies on the effect of the time of occlusion on the joint position sense must be highlighted.

## Figures and Tables

**Table 1:** Characteristics of the participants of the study.

	BFR Group	Placebo Group	Control Group	*p*
*N*	20	20	20	n/a
Males/females	10/10	10/10	10/10	n/a
Age (years)	22.70 ± 0.80	22.70 ± 1.49	23.40 ± 1.31	0.128
Body mass (kg)	72.85 ± 14	70.40 ± 15.30	68.90 ± 11.08	0.660
Body height (m)	1.76 ± 0.08	1.73 ± 0.09	1.75 ± 0.08	0.354
BMI (kg/m^2^)	23.28 ± 3.50	23.41 ± 4.40	22.43 ± 2.50	0.571

Values are expressed as arithmetic mean and standard deviation (±). BFR group, the group receiving blood flow restriction; control group, the group receiving no intervention; *n*, number of participants; *p*, the value of statistical significance; placebo group, the group receiving an inactive intervention.

**Table 2:** The comparison of values of mean absolute angular error for the target position of a 45° wrist extension obtained during the first and the second wrist joint position sense measurements.

Mean Absolute Angular Error for a 45° Wrist Extension (°)				
Studied Group	Studied Limb	1st Measurement	2nd Measurement	*p*
BFR Group	Right	7.19 (5.11, 9.28)	8.44 (5.94, 10.93)	0.336
	Left	6.62 (5.06, 8.18)	8.84 (5.57, 12.12)	0.271
Placebo Group	Right	8.16 (6.48, 9.85)	6.88 (5.03, 8.72)	0.311
	Left	7.94 (6.38, 9.49)	6.85 (4.59, 9.11)	0.477
Control Group	Right	10.92 (7.07, 14.76)	9.62 (6.22, 13.01)	0.355
	Left	6.62 (4.88, 8.35)	5.69 (4.24, 7.13)	0.093

Values are expressed as arithmetic mean and 95% confidence interval (±). BFR group, the group receiving blood flow restriction; control group, the group receiving no intervention; *p*, the statistical significance value; placebo group, the group receiving an inactive intervention.

**Table 3:** The comparison of values of mean absolute angular error for the target position of a 30° wrist flexion obtained during the first and the second wrist joint position sense measurements.

Mean Absolute Angular Error for a 30° Wrist Flexion (°)				
Studied Group	Studied Limb	1st Measurement	2nd Measurement	*p*
BFR Group	Right	11.28 (7.79, 14.77)	16.88 (12.86, 20.91)	**0.011**
	Left	11.51 (9.77, 13.25)	16.21 (12.89, 19.52)	**0.019**
Placebo Group	Right	12.46 (9.35, 15.56)	12.35 (9.61, 15.09)	0.948
	Left	13.22 (10.39, 16.04)	11.61 (9.19, 14.03)	0.241
Control Group	Right	11.04 (7.67, 14.40)	11.28 (7.96, 14.16)	0.647
	Left	13.74 (8.67, 18.81)	13.24 (9.61, 16.87)	0.904

Values expressed as arithmetic mean and 95% confidence interval (±). BFR group, the group receiving blood flow restriction; control group, the group receiving no intervention; *p*, the statistical significance value; placebo group, the group receiving an inactive intervention. The *p* < 0.05 is written in bold.

## Data Availability

The data presented in this study are available at a reasonable request from the corresponding author.
